# Supplementary material for: The small molecule ISRIB rescues the stability and activity of Vanishing White Matter Disease eIF2B mutant complexes
Source: eLife. 2018 Feb 28;7:e32733. doi: 10.7554/eLife.32733 (PMC5829914; doi:10.7554/eLife.32733)
Supplement: Supplementary file 1. [file elife-32733-supp1.docx]

**Supplementary File 1. Allele sequences of HEK293T mutant cell lines used in this study**

| **eIF2B subunit** | **Mutation** | **Clone ID** | **Alleles sequenced by TOPO cloning** |
| --- | --- | --- | --- |
| **α** | V183F | bC5 | Point mutation in V183F; silent mutation in PAM sequence  5-actgtggtgctagatgctgct**T**tcgg**A**tga-3  3-tgacaccacgatctacgacga**A**agcc**T**act-5 |
|  |  |  | Point mutation in V183F  5-actgtggtgctagatgctgct**T**tcgggtga-3  3-tgacaccacgatctacgacga**A**agcccact-5 |
|  |  | cA1 | Point mutation in V183F; silent mutation in PAM sequence  5-actgtggtgctagatgctgct**T**tcgg**A**tga-3  3-tgacaccacgatctacgacga**A**agcc**T**act-5 |
|  |  |  | Point mutation in V183F  5-actgtggtgctagatgctgct**T**tcgggtga-3  3-tgacaccacgatctacgacga**A**agcccact-5 |
| **γ** | I346T | bE3 | Point mutation in I346T, silent mutation in PAM sequence  5-ccaccagt**G**cattcgtcagcccaga**C**tgtcag-3  3-ggtggtca**C**gtaagcagtcgggtct**G**acagtc-5 |
|  |  |  | 1 bp insertion; frameshift leads to stop codon at aa 355  5-ccaccagtccattcg**G**tcagcccagattgtca-3  3-ggtggtcaggtaagc**C**agtcgggtctaacagt-5 |
|  |  | D1* | Point mutation in I346T, silent mutation in PAM sequence  5-ccaccagt**G**cattcgtcagcccaga**C**tgtcag-3  3-ggtggtca**C**gtaagcagtcgggtct**G**acagtc-5 |
|  |  |  | 3 bp deletion  *Does not lead to early stop but instead deletes 1 residue (Ser); GEF defect is observed in this clone  5-gtccattcagcccagattgtcagcaaacacctg-3  3-caggtaagtcgggtctaacagtcgtttgtggac-5 |
|  |  |  | 12 bp deletion  *Does not lead to early stop but instead deletes 4 residues (Pro-Pro-Ala-Gln); GEF defect is observed for this clone  5-gcccagattgtcagcaaacacctg-3  3-cgggtctaacagtcgtttgtggac-5 |
| **δ** | R483W | G8 | Point mutation in R483W, silent mutation in PAM  5-cacgcatcTcta**T**ggttgttgaatctagtc-3  3-gtgcgtagAgat**A**ccaacaacttagatcag-5 |
|  |  |  | 1 bp insertion; frameshift leads to stop codon at aa 490  5-cacgcatccctac**C**ggttgttgaatctagtctatga-3  3-gtgcgtagggatg**G**ccaacaacttagatcagatact-5 |
|  |  |  | 2 bp substitutions; frameshift leads to stop codon at aa 524  5-cacgcatcccta**G**gg**A**tgttgaatctagtctat-3  3-gtgcgtagggat**C**cc**T**acaacttagatcagata-5 |
|  |  | A10 | Point mutation in R483W; silent mutation in PAM sequence  5-cacgcatc**T**cta**T**ggttgttgaatctagtc-3  3-gtgcgtag**A**gat**A**ccaacaacttagatcag-5 |
|  |  |  | Point mutation in R483W  5-cacgcatcccta**T**ggttgttgaatctagtc-3  3-gtgcgtagggat**A**ccaacaacttagatcag-5 |
| **ε** | R113H | H4 | Point mutation in R195; silent mutation in PAM sequence  5-tcaaagtggtgcc**A**ccctacatctctcaatgt**A**gttcgaat-3  3-agtttcaccacgg**T**gggatgtagagagttaca**T**caagctta-5 |
|  |  |  | 1 bp insertion; frameshift leads to stop codon at aa 137  5-gccctacatctctcaa**A**tgtggttcgaataattacatcagag-3  3-cgggatgtagagagtt**T**acaccaagcttattaatgtagtctc-5 |
|  |  | A5 | Point mutation in R113; silent mutation in PAM sequence  5-aaagtggtgcc**A**ccctacatctctcaatgt**T**gttcgaataat-3  3-tttcaccacgg**T**gggatgtagagagttaca**A**caagcttatta-5 |
|  |  |  | 1 bp insertion; frameshift leads to stop codon at aa 137  5-tacatctctcaa**A**tgtggttcgaataattacatcagagctct-3  3-atgtagagagtt**T**acaccaagcttattaatgtagtctcgaga-5 |
|  | R195H | G10 | Point mutation in R195; silent mutation in PAM sequence  5-agccacccaactc**A**ttgccacgaagacaatgt**C**gtagtggct-3  3-tcggtgggttgag**T**aacggtgcttctgttaca**G**catcaccga-5 |
|  |  |  | 1 bp insertion; frameshift leads to stop codon at aa 207  5-actcgttgccacgaagac**A**aatgtggtagtggctgtggatag-3  3-tgagcaacggtgcttctg**T**ttacaccatcaccgacacctatc-3 |
|  |  | aG11 | Point mutation in R195 (Homozygous)  5-agccacccaactc**A**ttgccacgaagacaatgtggtagtggct-3  3-tcggtgggttgag**T**aacggtgcttctgttacaccatcaccga-5 |
